# Unmanned Aerial Vehicle Photogrammetry for Monitoring the Geometric Changes of Reclaimed Landfills

**DOI:** 10.3390/s24227247

**Published:** 2024-11-13

**Authors:** Grzegorz Pasternak, Klaudia Pasternak, Eugeniusz Koda, Paweł Ogrodnik

**Affiliations:** 1 Institute of Civil Engineering, Warsaw University of Life Sciences—SGGW, 02-776 Warsaw, Poland; grzegorz_pasternak@sggw.edu.pl (G.P.); eugeniusz_koda@sggw.edu.pl (E.K.); 2 Department of Imagery Intelligence, Faculty of Civil Engineering and Geodesy, Military University of Technology (WAT), 00-908 Warsaw, Poland; klaudia.pasternak@wat.edu.pl

**Keywords:** UAV, landfill, monitoring, photogrammetry, settlement, reclamation

## Abstract

Monitoring reclaimed landfills is essential for ensuring their stability and monitoring the regularity of facility settlement. Insufficient recognition of the magnitude and directions of these changes can lead to serious damage to the body of the landfill (landslides, sinkholes) and, consequently, threaten the environment and the life and health of people near landfills. This study focuses on using UAV photogrammetry to monitor geometric changes in reclaimed landfills. This approach highlights the advantages of UAVs in expanding the monitoring and providing precise information critical for decision-making in the reclamation process. This study presents the result of annual photogrammetry measurements at the Słabomierz–Krzyżówka reclaimed landfill, located in the central part of Poland. The Multiscale Model to Model Cloud Comparison (M3C2) algorithm was used to determine deformation at the landfill. The results were simultaneously compared with the landfill’s reference (angular–linear) measurements. The mean vertical displacement error determined by the photogrammetric method was ±2.3 cm. The results showed that, with an appropriate measurement methodology, it is possible to decide on changes in geometry reliably. The collected 3D data also gives the possibility to improve the decision-making process related to repairing damage or determining the reclamation direction of the landfill, as well as preparing further development plans.

## 1. Introduction

According to 2022 data from the Statistics Poland (GUS), there are 259 active landfills and more than 600 landfills that have been closed and partially or fully reclaimed. The 1999 European Union (EU) Landfill Directive and the EU’s overall policy of sustainable waste management calls for a gradual reduction in the amount of municipal waste sent to landfills. It also imposes technical and environmental requirements that landfills must meet, which will indirectly lead to the closure and reclamation of old landfills that do not meet environmental standards and the creation of new landfills that meet these standards [1,2]. As a result, it is recognized that the number of reclaimed landfills will continue to increase in the coming years. Municipal landfills are usually located in close proximity to large cities. The growth in the size of cities results in the integration of landfills into their urban fabric over time. Often, in such cases, as compensation for the landfill’s long-standing negative impacts on the immediate neighborhood (odor, fowl), it is transformed into public facilities with recreational, park, sports, museum, or exhibition functions [3]. The use of facilities in the context of electricity production (biogas plant, photovoltaic farm, wind farms) is also a common case. An example of such a landfill is the Słabomierz–Krzyżówka landfill, the future post-reclamation development of which is intended for a photovoltaic farm. This example was used as a case study in this paper.

Monitoring reclaimed landfills is crucial due to potential damage resulting from insufficient compaction of waste layers and inadequate slope protection. Landfills are geotechnical structures that can be structurally compared to earth structures made of anthropogenic materials equipped with drains, reinforcements, and seals. The large surface area, volume, and thickness of landfills, and the heterogeneity of the landfilled waste lead to the need for periodic monitoring of the landfills [4,5,6]. The minimum frequency of landfill surveying is set out in the Regulation of the Minister of the Environment of 30 April 2013 [7] and depends on the phase in which the landfill is currently located. Monitoring should be carried out every three months during the operational phase and every 12 months during the post-operational phase. The Regulation [7] defines monitoring as the control of subsidence of the surface of a facility by geodetic methods based on measurements of displacements of geodetic points stabilized on the landfill’s surface. The monitoring should also include an assessment of slope stability based on geotechnical methods. Early detection of displaced materials within the landfill is a critical issue in early warnings of a landslide. With geodetic measurements, it is possible to ensure landfills’ geotechnical safety by monitoring site settlement’s regularity. Based on long-term measurements, the directions of future displacements and deformations of the landfill body can be modeled and predicted [8,9,10,11,12].

Landfill monitoring is performed using both classical and modern survey techniques. Classical measurements include total station, leveling, and Global Navigation Satellite Systems (GNSS) measurements. However, these measurements only provide point data on changes in the geometry of geotechnical objects [8]. Laser scanning and photogrammetric techniques [13] make it possible to obtain a dense point cloud representing the area of the entire landfill. The point cloud can be obtained using photogrammetric techniques [14,15,16], Terrestrial Laser Scanning (TLS) [17,18], or Aerial Laser Scanning (also named Airborne Laser Scanning (ALS) [19]. Both TLS and Structure from Motion (SfM) are used in the inventory of underground caves [20,21], mountain landslides [22], slope stability [19], open-pit mining hazards [23], and construction monitoring [24,25].

Low-altitude point clouds can be acquired from aircraft and Unmanned Aerial Vehicle (UAV). The use of UAVs allows one to make non-invasive measurements of the geometry of the entire landfill, which is confirmed by numerous publications [14,26]. As a result, the UAV operator is not directly exposed to pollution associated with leachate or biogas. The use of the photogrammetric method with a UAV is a cheaper alternative to laser scanning (aerial and ground-based). Data acquired from the ground or low-altitudes are most often used for monitoring the condition of structures, object inventory, volume determination, vegetation cover analysis (monitoring vegetation growth and detecting areas of vegetation dieback) [27], and in the process of designing and planning changes in the development of a given area [28]. Based on UAV data, it is possible to obtain products, i.e., orthophotomaps, 3D models, digital terrain models (DTM), or digital surface models (DSM) [29,30,31,32,33]. These products can be applied for other purposes in addition to monitoring site geometry. For example, a landfill’s DTM can be used to detect and monitor biogas [34]. Based on sensors, i.e., thermal imaging cameras, landfills can also be monitored. Thermal sensors have the ability to measure the temperature of the indicated object. Thermal imagery can be used to prevent leaks or fires in dumps or yards. In such cases, observation of the area from a low ceiling is possible in real-time [35,36].

Monitoring landfills is also crucial for public purposes in the context of their subsequent development. Such sites are often converted into sports or recreational facilities. An example is a recreational and winter park with an accessible slope for tobogganers and skiers (in winter) and for joggers and cyclists (in summer). Another instance might be the redevelopment of a landfill into a photovoltaic or wind farm. The possibility of converting a landfill into a public facility is contingent on the results of geotechnical studies obtained and analyses related to sanitary conditions. Meeting the requirements for the safe foundation of the planned construction facilities is a requirement for starting design work [37,38,39].

The aim of this paper was to present the possibility of using the photogrammetric method to monitor the geometry of reclaimed landfills, using the Słabomierz–Krzyżówka landfill as a case. The results of the measurements obtained from the photogrammetric method were related to the reference data obtained from linear–angular measurements from trigonometric leveling. This paper presents the possibilities and limitations of using the photogrammetric method in landfill monitoring. For the authors, the photogrammetric method can be an interesting and low-cost alternative in acquiring geometric data about landfills as well as in the context of geometric changes caused by subsidence. The paper also presents instances of damage caused by surface run-off. Nowadays, monitoring is carried out at the Słabomierz–Krzyżówka landfill using classical methods (trigonometric leveling and GNSS).

## 2. Materials and Methods

This section presents the characteristics of the study area (Section 2.1) and the measurement equipment used (Section 2.2). It also describes the proposed method for determining displacements based on measurements made by the photogrammetric method (Section 2.2).

### 2.1. Study Area

**Figure 1:**
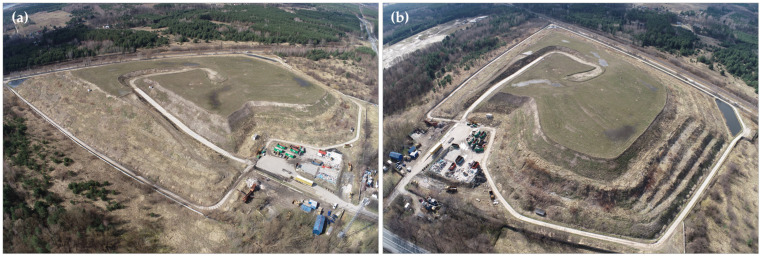
View of the Słabomierz–Krzyżówka landfill from a (**a**) south-east and (**b**) north-east direction.

The Słabomierz–Krzyżówka landfill is located in the Żyrardowski district of Mazowieckie province (Poland) at the site of an old sand mining pit. From 1970 to 1992, municipal and industrial waste from the Żyrardów city area was deposited at the landfill. Later, non-segregated municipal waste was deposited. The landfill with technical facilities currently covers an area of 14.2 hectares, and the landfill itself covers an area of 8.7 hectares (Figure 1).

The Słabomierz–Krzyżówka landfill was closed in 2022. The site is to be converted into a photovoltaic farm with an elevation at the crown of the object of 175 m above sea level (27 m from the foot of the landfill). An installation has been built at the landfill to extract and analyze the composition of biogas, which is produced by the biodegradation process of waste [40,41].

Groundwater quality is monitored at the landfill and in its vicinity using four piezometers. Leachate emitted from the landfill is collected by a band drain located at the bottom of the facility’s slopes. The body of the landfill is surrounded by a vertical curtain to prevent contaminants from entering neighboring areas.

The landfill is partially and to a different degree covered with vegetation. The landfill carries out maintenance work consisting of periodic cutting of grass located on the crown of the landfill and slope shelves.

At the landfill, 15 benchmarks have been located to monitor the facility’s subsidence.

### 2.2. Methodology

Monitoring of the landfill is carried out periodically once a year using line-angle and GNSS measurements. Due to the vegetation present at the landfill, photogrammetric measurements were taken in early spring (March). This enabled the maximum elimination of the influence of vegetation on the results of deformation determination by this method. At the same time, control measurements were carried out at the landfill using the classic method (measurement of control points using the total station method). The measurements were made using a Topcon (Tokyo, Japan) GPT-9003M total station. Measurements were referenced to a network of control points located outside the landfill’s area of influence. The measured angle-linear network was aligned using the least-squares method. The average error of the situational position of the points was ±1.1 cm (Set 1) and 1.0 cm (Set 2), and a height position error of ±0.5 cm (Set 1) and ±0.3 cm (Set 2). Two measurement series were made in March 2023 and March 2024.

Photogrammetric measurements were made using a DJI (Shenzhen, China) Phantom 4 RTK platform equipped with a 1-inch 20-megapixel CMOS sensor. In order to generate a dense point cloud of the research object, both nadir and oblique images were taken. The images were taken at altitudes of 50 and 80 m AGL, respectively. For photogrammetric raids, in order to increase the accuracy of height measurement, it is recommended to carry out a second raid with a variable flight altitude or to increase the number of control points and their placement, taking into account as much altitude variation as possible. This will reduce correlations of unknowns estimated during aerotriangulation [42]. The authors’ purpose was to obtain an average GSD < 3 cm. For this reason, a flight was made at 50 m and a second one (to increase the accuracy of the height measurement) at 80 m. Detailed specifications of the acquired data and the resulting point clouds are shown in Table 1.

**Figure 2:**
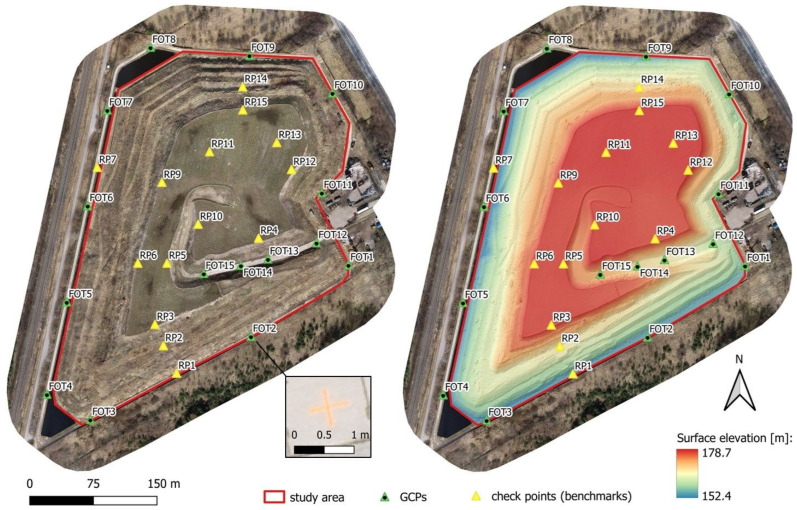
Location of GCPs on the generated orthophotomap of the landfill (**left**) and on the DSM (**right**).

For georeferencing, 15 Ground Control Points (GCPs) and 14 Check Points were established, the locations of which are shown in Figure 2.

Control points in the photogrammetric method should be distributed evenly and at different heights over the entire area of the surveyed object. For linear objects, control points should be further densified. The control points were placed at the corners of the block, along the extreme rows, and at the beginnings and ends of the selected rows. Points located on the crown and slopes of the landfill slopes were selected as check points. Check points were not involved in the final alignment; they were used to assess accuracy. The position of these points was determined using a line–angular network by total station measurements. The monitoring of individual control points (benchmarks) located on the body of the landfill often does not reflect the actual changes occurring in the area of the entire site. It is assumed that the monitored points are representative of the entire analyzed object. A denser network of points more accurately approximates the distribution of displacements but results in increased costs and time-consuming measurements. Alternatives to these measurements are surface monitoring methods of the surveyed object, such as the laser scanning method (ground and aerial) and the photogrammetric method, which are based on the measurement of unstabilized points—directly measuring the ground. These methods have been successfully used in periodic geodetic monitoring of facilities with structures similar to landfills. These include open-pit mines, natural landslide slopes, post-production landfills, and various types of bulk product storage areas. In the publication [43], Pasternak G. et al. compared 3 types of measurement methods, comparing them in terms of data acquisition time, results processing time, number of operators required to perform the measurement, and estimated costs. The study showed that the photogrammetric method is a promising method in the context of monitoring sites not covered with vegetation or covered with low vegetation (such vegetation is present in most of the area on the Słabomierz–Krzyżówka landfill). It is necessary to apply the appropriate method to the facility. In addition, this method is a low-cost method compared to the method of scanning with UAV or ground scanning while maintaining a high accuracy of measurement. Due to the conditions at the Słabomierz–Krzyżówka landfill, such as low vegetation and open terrain (no aerial obstructions), it was possible to use the photogrammetric method.

In order to validate the applied measurement methodology (photogrammetric method), 15 control points (control points) were established on the site, the displacements of which were measured using the linear–angular method (reference method).

The longitudinal coverage of the nadir images was 90%, and the transverse coverage was 80%. In oblique images, the coverage was 70 and 60%, respectively. A total of 1621 images were acquired in Set 1 and 1166 images in Set 2.

Processing of the acquired photogrammetric data was performed in the Pix4D’s (Lausanne, Switzerland) Pix4Dmapper version 4.7.5 software. The processing used the parameters listed in Table 2.

The interior and exterior orientation elements of each image were determined, generating the tie points. The primary purpose of digital aerotriangulation is to determine the elements of external orientation and to relate field coordinates of control points and image data according to Equations (1) and (2):

(1) $$x_{k}-x_{0}=-f_{k}\left[\frac{r_{11}\left(X_{T}-X_{kr}\right)+r_{21}\left(Y_{T}-Y_{kr}\right)+r_{31}\left(Z_{T}-Z_{kr}\right)}{r_{13}\left(X_{T}-X_{kr}\right)+r_{23}\left(Y_{T}-Y_{kr}\right)+r_{33}\left(Z_{T}-Z_{kr}\right)}\right],$$

(2) $$y_{k}-y_{0}=-f_{k}\left[\frac{r_{12}\left(X_{T}-X_{kr}\right)+r_{22}\left(Y_{T}-Y_{kr}\right)+r_{32}\left(Z_{T}-Z_{kr}\right)}{r_{13}\left(X_{T}-X_{kr}\right)+r_{23}\left(Y_{T}-Y_{kr}\right)+r_{33}\left(Z_{T}-Z_{kr}\right)}\right],$$

where

*x_k_*, *y_k_*—control point image coordinates,
*x*_0_, *y*_0_—coordinates of the camera principal point,
*f_k_*—focal length,
*X_T_*, *Y_T_*, *Z_T_*—ground coordinates of control point,
*X_kr_*, *Y_kr_*, *Z_kr_*—coordinates of the center of the camera projection in the ground system.

Proper georeferencing requires knowledge of GPS/INS data and the location of field matrix points. These data make it possible to integrate the measured centers of projections using the GPS technique with the field measurements of the matrix points by including additional parameters in the georeferencing optimization process.

**Figure 3:**
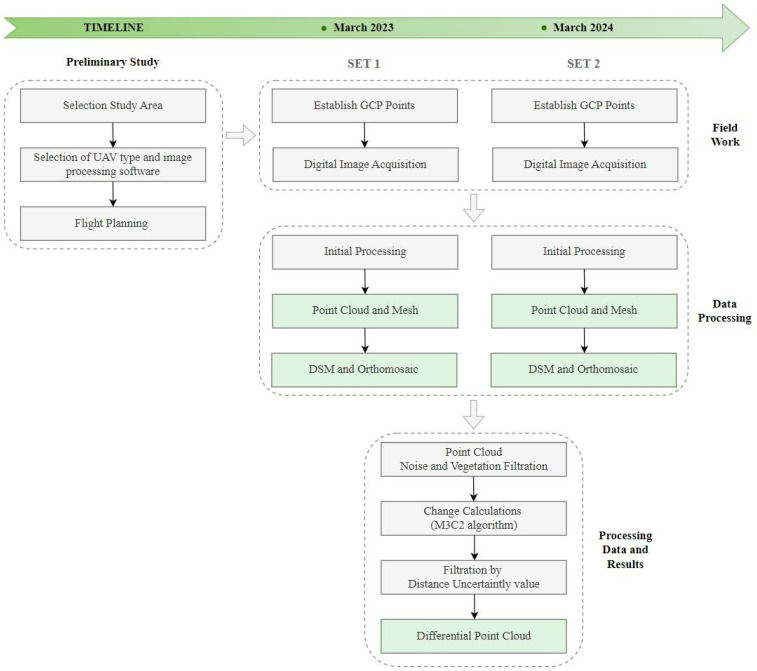
Flowchart of research methodology.

Based on the photogrammetric block alignment, a GSD of 1.84 cm (Set 1) and 2.57 cm (Set 2) was obtained. A dense point cloud was generated using multi-image correlation (Dense Matching). The average density of the point cloud was 412 points/1 m^3^ (Set 1) and 229 points/1 m^3^ (Set 2). The RMSE error for both Set 1 and Set 2 was ±7 mm. The photogrammetric data acquisition and processing flowchart with the main steps is shown in Figure 3.

Specific work plan about full scope of research is as follows:

Preliminary Study

1. Selection Study Area: Słabomierz–Krzyżówka Landfill;
2. Selection of UAV type (DJI Phantom 4 RTK) and image processing software (Pix4Dmapper);
3. Flight Planning, including location of GCP and check points;
  3.1. Plan the height of the photogrammetric flight based on the GSD < 3 cm;
  3.2. Plan the front and side coverage of the digital image block;
  3.3. Determine the flight path and camera parameters (ISO, shutter speed, aperture);
  3.4. Design the number and distribution of control points and check points, taking into account as much elevation variation as possible;

Field Work

4. Establish and measure GCPs using a line–angular network by total station measurements;
5. Digital Image Acquisition;
  5.1. Nadir images (AGL = 50 m, front image overlap = 90%, side image overlap = 80%, camera angle = 90°);
  5.2. Oblique images (AGL = 80 m, front image overlap = 70%, side image overlap = 60%, camera angle = 60°) acquired in four different directions (north, south, west, east);

Data Processing

6. Initial Processing;
7. Aerotriangulation with verification of control photopoints;
8. Point Cloud and Mesh generation using multi-image correlation (dense matching);
9. DSM and Orthomosaic generation;
10. Point Cloud Noise and Vegetation Filtration using Cloth Simulation Filtering (CSF) algorithm;
11. Change Calculations using M3C2 algorithm.

In addition to the point cloud, a Mesh model, DSM, and orthophotomap of the landfill area were generated for each data set (Set 1 and 2). A crucial stage was point cloud filtration due to the large vegetation, especially in the slope area of the landfill. Cloth Simulation Filtering (CSF) was used to extract the ground layer, which was used for further analyses to determine the deformation of the landfill [44,45,46,47]. The CSF filter is a point cloud filtering algorithm designed to separate ground and non-ground points in the point cloud. This filtration is based on virtual Cloth Simulation. It is a simulation of the interactions between the point cloud points and corresponding cloth nodes, whose location is determined to generate an approximation of the ground points. The ground points are extracted by comparing point cloud points and the generated surface [48].

**Figure 4:**
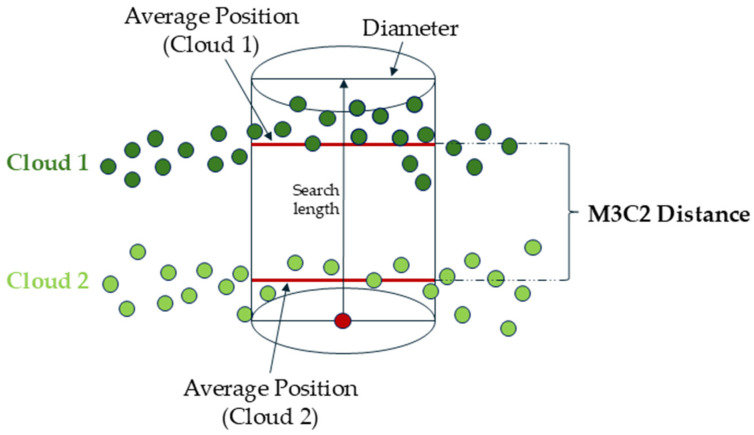
M3C2 algorithm functional rule.

Based on the obtained point clouds (from measurement campaigns 1 and 2), the differences between them were determined using the Multiscale Model to Model Cloud Comparison (M3C2) method [49,50]. The M3C2 algorithm was used to determine the deformation of the landfill body by comparing photogrammetric data acquired in two survey campaigns. To validate the accuracy of the change detection calculations, these results were compared with direct measurements (linear–angular measurements). The obtained results show the annual deformations of the landfill. There are several approaches to detecting surface changes. These are methods such as DEM differentiation, Cloud to Cloud (C2C), Cloud to Mesh (C2M), or M3C2. Each of these algorithms is applied to different types of data, depending on the purpose. According to the authors [51,52,53,54], for point clouds obtained from a photogrammetric method with high noise, the preferred method for change detection is the M3C2 method. Therefore, this method was used in the current research. The M3C2 algorithm determines the distance along the local normal vector, which is estimated based on the vicinity of each point. The method considers the local orientation of the surface in the distance calculation process. The general rule of the algorithm is based on developing search cylinders along normal vectors to locally average the changes between two point clouds, as shown in Figure 4.

The radius of the cylinder was determined empirically and was 25 cm. The M3C2 method offers a reliable change detection procedure that is applied directly to point clouds (differently from other methods, i.e., C2C).

**Figure 5:**
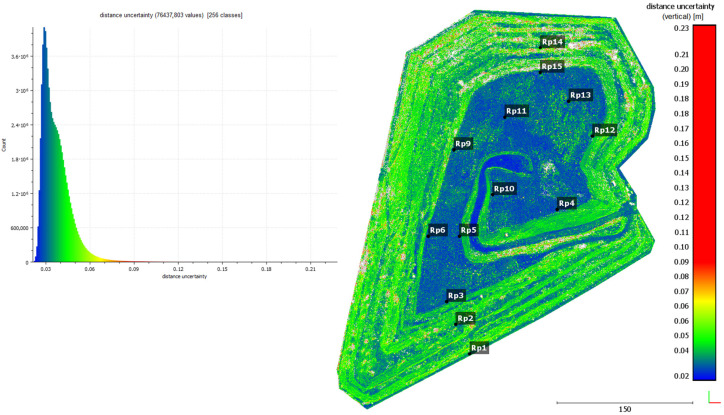
Distance Uncertainty calculated by the M3C2 algorithm.

For each estimated distance, the M3C2 algorithm also allowed for computing the Distance Uncertainty. Distance Uncertainty in the M3C2 algorithm refers to the uncertainty in estimating the distance between two point clouds. This value is due to the measurement accuracy of the measuring device, the accuracy of the mutual registration of point clouds, and the roughness of the point clouds. In the M3C2 algorithm, the distance uncertainty is calculated based on the propagation of errors associated with the measurement of points, their capture, and interpolation of normals to surfaces. This value allows a reliable assessment of whether the measured distances between point clouds are relevant in the context of surface change analysis. The value of the distance uncertainty is used to assess whether the detected change is real or may be due to measurement errors. If the distance between points is less than the calculated uncertainty, the change may be considered unreliable and may be eliminated from further analysis. The result of the Distance Uncertainty calculation is shown in Figure 5.

In Figure 5, it can be seen that increased Distance Uncertainty occurs mainly in the area of slopes covered with higher vegetation. Despite the filtering of vegetation, the point cloud in these areas is characterized by increased roughness, which affects the results of displacement calculations. In order to increase the reliability of the displacement results, the point cloud was subjected to filtering based on distance uncertainty. This value was determined empirically, and it was set as 5 cm (limit value).

## 3. Results

**Figure 6:**
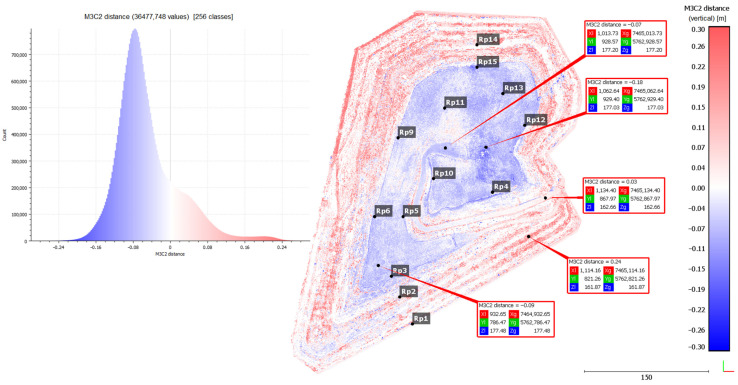
Differential point cloud showing vertical displacements of the Słabomierz–Krzyżówka landfill body.

The analyses resulted in a differential point cloud showing vertical displacements of the Słabomierz–Krzyżówka landfill body (Figure 6). In Figure 6, the subsidence is shown in blue, and the uplifts in red. The white color represents the points without changes. It can be pointed out that subsidence occurs mainly on the crown of the landfill and, for the most part, has a value in the range of −5 to −15 cm. The exceptions are two areas. The first is dark blue, with increased subsidence, reaching up to −20 cm, located in the central part of the crown, and the second is white and light red, showing a slight uplift, located near the Rp11 point. The first location is in the region of the lowered area (basin) where rainwater collects. Increased subsidence values in this area can, therefore, be caused by both soil leaching and compaction caused by the weight of collected rainwater. The second location is where workers dumped soil collected during repair work, which was detected during the field interview and confirmed on the orthophotomap of the study area. Adding to this causes the formation of fake uplifts in this area. The uplifts occur mainly on the slopes of the landfill and take values of up to 25 cm. They are caused by the pushing out of the soil by the self-weight of the landfill.

For the purpose of verification of the propriety of the determined displacements, the results of the analyses were compared with the displacements determined on the control points (benchmarks). Differences in the determined displacements by both methods were determined. The average value of these differences was 0.014 m. It can be pointed out that the main differences in the results of the two methods are in areas with high and medium vegetation (Table 3).

The mean vertical deformation error using the photogrammetric method can be determined from the formula:

(3) $$m∆Z=\pm \sqrt{{{mZ}_{Set2}}^{2}+{\left(-{mZ}_{Set1}\right)}^{2}},$$

where

m∆Z—mean vertical displacement error,
mZ_Set1_—mean height error at check points (Set 1),
mZ_Set2_—mean height error at check points (Set 2).

The mean vertical deformation error using the photogrammetric method was ±2.3 cm.

**Figure 7:**
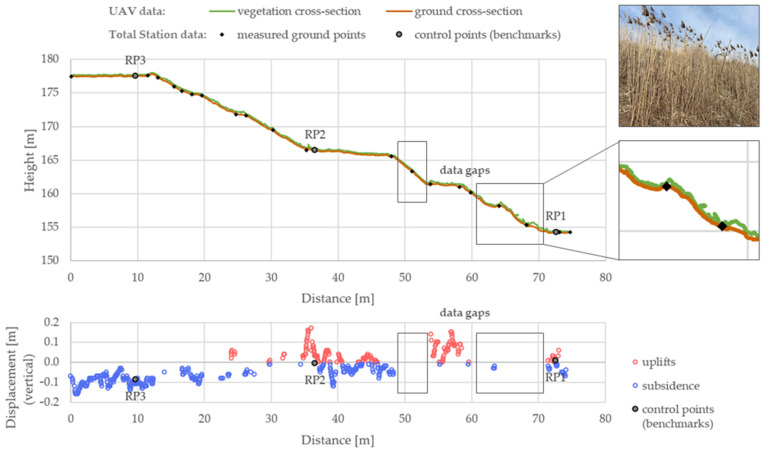
Vertical cross-section (**up**) and vertical displacements of the slope (**down**).

To validate the results, a vertical cross-section was also made through one of the slopes that are being monitored geotechnically. There are three control points (benchmarks) on this slope (RP1, RP2, RP3), the position of which is shown in the graph (Figure 7). This graph shows data acquired from the UAV (Set 2) and reference data (from total station). The graph below shows the vertical displacements calculated by the M3C2 method from the UAV-acquired data (marked as blue and red circles) and the vertical displacements of the control points—reference data (marked as black circles).

Analyzing the graphs above, it can be seen that the slope settles in the upper part and uplifts in the lower parts. This result is consistent with the geotechnical predictions. It is caused by the gradual compaction of the soil and stored waste, its biodegradation, and mechanical creep caused by the dead weight of the landfill. These results are also consistent with reference data—displacements determined by the total station method.

Due to the high vegetation, measurements at the landfill were made in early spring. However, this did not entirely eliminate the impact of vegetation on the measurements due to the lack of maintenance treatments on the slopes of the landfill. After the winter period, dry parts of grasses and shrubs are present on the slopes, making it impossible to measure the ground effectively using the photogrammetric method. The result of these limitations is a lack of displacement data (data gaps) in the areas with the highest vegetation. Points from these areas have been removed by the filtration process.

**Figure 8:**
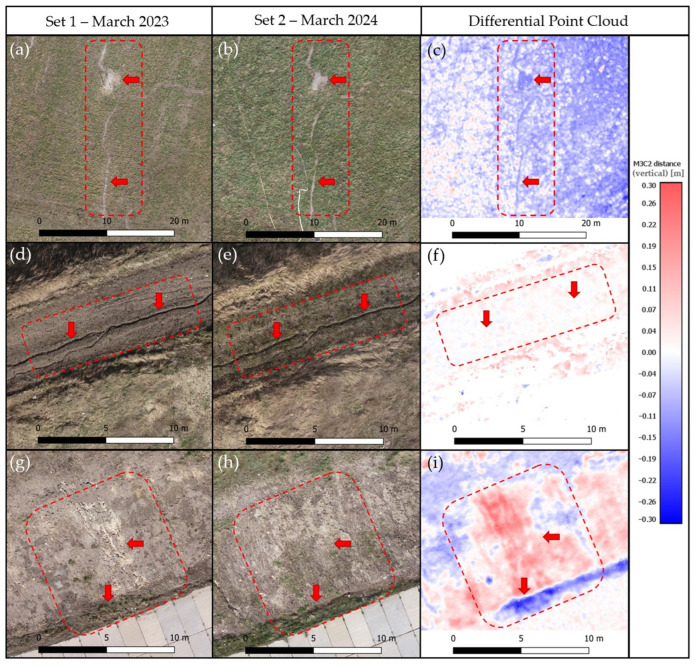
Examples of damage caused by surface runoff of rainwater on orthophotomap—Set 1: (**a**,**d**,**g**);—Set 2: (**b**,**e**,**h**), and on differential point cloud: (**c**,**f**,**i**).

Based on photogrammetric data, an orthophotomap of the landfill area was also generated. These data can be applied in the visual detection of landfill geometry changes, i.e., cracks, landslides, wildlife damage, or soil leaching by surface water runoff. Figure 8 shows three examples of damage to the landfill’s lump, as detected by the acquired photogrammetric data.

Figure 8a–c shows soil leaching caused by surface water runoff. This area is on the crown of the landfill. The red dashed line highlights the area of change, while areas of particular importance are indicated with an arrow. Based only on the interpretation of photogrammetric images, it is impossible to assess the changes in the area. Interpretation of this is possible only after analyzing the differential point cloud. In Figure 8c, it can be observed that the land inside the funnel has decreased by a larger value than the land around the funnel. This indicates the slow leaching of soil from the area. Figure 8d–f also shows a funnel formed by surface water runoff. However, no changes are visible on the differential point cloud, indicating that at the time of monitoring the landfill, the process of soil leaching did not occur in this area. This problem was pointed out even before the introduction of photogrammetric monitoring. An appropriate remediation plan against water runoff has been put into place in the field. Figure 8g–i also shows damage caused by surface water runoff. Figure 8g shows the gouges in the ground of the slope (highlighted by the red arrow). The slope has been repaired, as shown in Figure 8h. Drainage was also made in the area to remove water. Figure 8i shows the uplift (marked in red) caused by reinforcement through the incorporation of soil leached during rain. A large soil cavity is also visible at the base of the slope, which was leached and then erased.

## 4. Discussion

The research provides an overview of the possibilities of monitoring landfills using the photogrammetric method. One of the main advantages of this method is its non-invasiveness. The operator, while performing a photogrammetric flight, is not exposed to contaminants associated with biogas and leachate escaping [14,26]. Choosing the photogrammetric method is a more economically achievable solution due to the lower price of the equipment compared to the Light Detection and Ranging (LiDAR) sensor [43]. However, it should be noted that the photogrammetric method has some limitations. One of its disadvantages is the inability to penetrate vegetation as much as with LiDAR data [19]. This is associated with the capture of consecutive reflected laser beams from the surface of objects (i.e., the landfill’s ground). In the case of dense vegetation, reliably filtering out this vegetation and determining object displacement is a difficult issue. If high vegetation is present, the preferred method is LiDAR, which allows vegetation penetration and direct measurement of the ground. If low vegetation is absent or ascendant, the photogrammetric method can be used. Recommendations for overcoming vegetation interference are as follows:

- Measurements should be taken on a windless day;
- There should be increased coverage of the images;
- Measurements should be taken when there is a low vegetation condition (early spring);
- There should be maintenance work before winter to eliminate vegetation.

To perform photogrammetric measurements, adequate knowledge of operators is required in planning flight surveys and in processing the results of these measurements to achieve adequate reliability and accuracy. Those performing the measurement must be properly trained and licensed. Another limitation of the photogrammetric method is the need for an adequate number of control points and check points, which must be located and measured before the flight is carried out. In the photogrammetric method, the accuracy of the model depends on the number and distribution of control points. To ensure adequate accuracy, a large number of control points is necessary, which increases the time and cost of field work. Measurements using the photogrammetric method are characterized by sensitivity to lighting conditions. The quality of photogrammetric images deteriorates when the images are underexposed, during haze or precipitation. These factors reduce the photo-interpretation possibilities in the acquired images.

Determination of the deformation of the landfill body by the Multiscale Model to Model Cloud Comparison (M3C2) method made it possible to confirm existing studies in this field [51,52,53,54]. Verification of the achieved results was made based on data from linear–angular measurements. According to the authors [51,52,53,54], the M3C2 method is the best method for detecting changes in case of data with high noise, which was confirmed by the results obtained in this article. The implementation of filtering by distance uncertainty value made it possible to increase the degree of certainty in the process of determining deformations between point clouds captured at different times. This filtering made it possible to eliminate sets of points characterized by high roughness, especially in the area of vegetation, i.e., on the slopes of the landfill. As a result of this process, the reliability of the obtained results of the object’s displacement has increased. Due to the growing seasons of vegetation, geodetic monitoring of landfills should be carried out in late autumn (late October/early November) or early spring (late March/early April) [43].

In addition to determining the deformation of an object, this article also shows other possibilities and advantages of the photogrammetric method. The generated orthophoto of the landfill allows the users to observe cracks or landslides, which are sometimes directly inaccessible to them. Monitoring this type of damage allows for preventive and quantitative landslide risk assessment and risk management. The acquired images are suitable for planning appropriate reinforcement solutions on the slopes of the landfill. The technological development of UAVs has meant that the use of these sensors in displacement monitoring and landslide hazard assessment has increased significantly.

## 5. Conclusions

The article presents the results obtained from photogrammetric measurements on the case of the reclaimed landfill Słabomierz–Krzyżówka. Based on the generated dense point clouds from the two measurement campaigns (March 2023 and March 2024), the differences between them were determined using the Multiscale Model to Model Cloud Comparison method. The result was the vertical displacement of the landfill body. Based on the analyses, it was concluded that subsidence occurred mainly on the crown of the landfill, and its magnitude reached a maximum of −20 cm. The verification of the analyses carried out was the determination of differences in the determined displacements at the control points. The average value of the differences obtained was 1.4 cm, and the major differences were obtained in the area of medium and high vegetation.

The results made it possible to determine displacements in the entire area of the landfill, not just at selected measurement points. This provides a wider overview of the deformation occurring at the landfill. This method can be used as a complement to classical geodetic monitoring. With the additional information, we can schedule future development of the site, as well as determine areas of potential risk associated with damage. This information can improve the decision-making process, related, for example, to the establishment of new controlled points (benchmarks) in areas of increased subsidence.

Photogrammetric monitoring, due to its low cost, can be repeated at high frequency—as needed and performed at high spatial resolution (GSD = 1.84 cm [Set 1] and 2.57 cm [Set 2] in this research). Simultaneously, it is a method that currently gives high accuracy (The mean vertical displacement error determined by the photogrammetric method was ±2.3 cm). The average error in the position of the control points was 1.5 cm in both Set 1 and Set 2. Additional products in the form of a mesh model and orthophotos, on the other hand, allow for a visual assessment of the facility and the detection of potential problems before they become major threats.

The disadvantage of the photogrammetric method is its inability to penetrate vegetation, which can be a problem with dense vegetation. These areas will lack information about the height of the land, or worse, the information will be incorrect. Insufficient or incorrect filtering of vegetation can lead to misinterpretations of displacement in these areas.

The results of the research using the photogrammetric method made it possible to analyze the deformation process of the selected research object (the Słabomierz–Krzyżówka landfill) and will also allow predictions of the behavior of this object in the future. The developed methodology can provide a solution for geotechnical engineers dealing with modeling and prediction of displacements occurring at reclaimed landfills.

## Figures and Tables

**Table 1:** Characteristics of the acquired data.

Data Set	Date	Images	GSD [cm]	GCPs	RMSE [m]		Average Point Cloud Density [per m^3^]	Check- Points	Check Points RMSE [m]	
					Error XY	Error Z			Error XY	Error Z
Set 1	March 2023	1621	1.84	15	0.006	0.008	412	14	0.011	0.017
Set 2	March 2024	1166	2.57	15	0.008	0.006	229	14	0.009	0.016

**Table 2:** Processing parameters setup.

Pix4Dmapper Parameters:	
Mode:	Standard 3D Maps
Initial processing:	Original image size for keypoints detection; Matching image pairs—aerial grid or corridor; Automatic target number of keypoints; Standard camera calibration method.
Point cloud and mesh:	Point cloud densification with half image size and optimal point density (minimum 3 matches); Medium resolution for 3D textured mesh.
DSM and orthomosaic:	Automatic resolution; DSM sharp noise filtering and surface smoothing.

**Table 3:** Comparison of displacements determined by photogrammetric and tachymetric methods.

Point No.	Vertical Displacements [m]			
	Leveling	UAV Photogrammetry	Difference	Vegetation
RP1	0.010	0.010	0.000	low
RP2	−0.005	0.040	0.045	high
RP3	−0.087	−0.110	−0.023	low
RP4	−0.138	−0.130	0.008	low
RP5	−0.083	−0.070	0.013	low
RP6	−0.089	−0.070	0.019	low
RP9	−0.106	−0.090	0.016	low
RP10	−0.094	−0.080	0.014	low
RP11	−0.101	−0.070	0.031	low
RP12	−0.148	−0.110	0.038	medium
RP13	−0.130	−0.110	0.020	low
RP14	−0.008	−0.010	−0.002	medium
RP15	−0.179	−0.170	0.009	low
Mean:			0.014	

## Data Availability

The data presented in this study are available on request from the co-author Grzegorz Pasternak, e-mail: grzegorz_pasternak@sggw.edu.pl.
